# Complex Regional Pain Syndrome With Central and Peripheral Nervous System Involvement in a Patient With Metastatic Lung Cancer

**DOI:** 10.7759/cureus.93304

**Published:** 2025-09-26

**Authors:** Sara S Amer, Daniel Cuadrao, Rishi Patel, Gizem B Keles, Kamal T Patel

**Affiliations:** 1 Department of Internal Medicine, Lake Erie College of Osteopathic Medicine, Tampa, USA; 2 Department of Cardiology, Lake Erie College of Osteopathic Medicine, Bradenton, USA; 3 College of Osteopathic Medicine, Lake Erie College of Osteopathic Medicine, Bradenton, USA; 4 Department of Medicine, Lake Erie College of Osteopathic Medicine, Tampa, USA; 5 Department of Pain Management, Tampa Bay Pain Associates, Lakeland, USA

**Keywords:** brain tumors (primary or brain metastasis), complex regional pain, interventional pain medicine, leg edema, metastatic lung

## Abstract

Complex regional pain syndrome (CRPS) is a chronic pain disorder characterized by sensory, motor, autonomic, and trophic disturbances, often following trauma, surgery, or neurologic injury. We present a diagnostically complex case with overlapping features of both type I and type II CRPS in a 64-year-old man with metastatic lung adenocarcinoma involving the central nervous system (CNS). The patient developed neuropathic pain, weakness, and edema in the left upper and lower extremities following trauma, radiation, and cervical and lumbosacral radiculopathies. Although his presentation met the Budapest diagnostic criteria for CRPS, diagnostic clarity was complicated by concurrent central (thalamic metastases) and peripheral (radiculopathy) nervous system pathology. Multimodal treatment, including physical therapy for desensitization and mobility, neuropathic medications such as gabapentin and duloxetine, sympathetic nerve blocks, and psychological support, was initiated and resulted in partial symptomatic improvement. This case highlights the importance of early recognition and a multidisciplinary approach in CRPS, particularly in patients with overlapping central and peripheral neurologic disease. Increased awareness of atypical presentations in oncology patients is essential to prevent diagnostic delays and to guide appropriate, individualized management.

## Introduction

Complex regional pain syndrome (CRPS) is a chronic neuropathic pain condition characterized by persistent and burning pain disproportionate to any known inciting event. It is classified into two types: type I, which occurs without identifiable nerve injury, and type II, which follows a confirmed nerve injury [1]. Although the exact pathophysiology remains unclear, CRPS is believed to involve a combination of peripheral and central nervous system (CNS) sensitization, autonomic dysfunction, and inflammatory processes [1]. This condition most commonly affects adults aged 30-60, with a higher prevalence in women, and frequently involves the upper extremities [1]. CRPS often arises after trauma, surgery, or fractures, but can also occur in the context of CNS lesions such as stroke, spinal cord injury, or brain tumors. Patients typically present with severe regional pain accompanied by sensory abnormalities (e.g., allodynia and hyperalgesia), vasomotor and sudomotor disturbances (e.g., skin color and temperature changes, and abnormal sweating), edema, motor dysfunction, and trophic changes that affect the skin, hair, or nails [2]. The diagnosis is clinical, guided by the Budapest criteria, which require specific patterns of signs and symptoms across sensory, vasomotor, sudomotor, and motor/trophic domains [1]. While imaging studies, such as bone scans or magnetic resonance imaging (MRI), may support the diagnosis, they are not definitive. Treatment involves a multidisciplinary approach, including physical therapy to preserve function; pharmacologic agents such as nonsteroidal anti-inflammatory drugs (NSAIDs), anticonvulsants, antidepressants, corticosteroids, and bisphosphonates; and interventional techniques such as sympathetic nerve blocks or spinal cord stimulation for refractory cases [2]. Early recognition and intervention are critical, as delayed treatment may lead to prolonged disability and chronic functional impairment. This case report describes a 64-year-old man who developed CRPS following a fall involving his upper extremities and radiation therapy for lung cancer.

## Case presentation

A 64-year-old man with a history of lung adenocarcinoma and brain metastases, status post-left upper lobectomy, durvalumab therapy, and stereotactic brain radiation to four intracranial lesions, came to the interventional pain management clinic. Ten years ago, the patient was diagnosed with a peripherally located adenocarcinoma of the left upper lobe, which was subsequently resected. The patient presents with progressive neuropathic pain and functional decline. He described burning, tingling, and stabbing pain localized to the left upper extremity (LUE), most severe in the shoulder and hand, and the left lower extremity (LLE), which began two years ago following a fall in which he landed on his arms. That same year, a worker’s compensation-related accident necessitated further imaging, and an MRI revealed four intracranial metastases: a right thalamic lesion measuring 1.7 cm, a right occipital lesion measuring 9 mm, a left frontal lesion measuring 6 mm, and a left parietal lesion measuring 2 mm, for which he further underwent three days of stereotactic brain radiation. His symptoms were exacerbated by subsequent radiation-induced left-sided numbness and visual field deficits of left homonymous hemianopia, attributed to metastatic brain lesions. Despite completing radiation, the left-sided sensory deficits were only partially resolved. In addition, the patient exhibited profound weakness, muscle twitching, reduced range of motion (ROM), and diffuse swelling of the LUE. He also reported decreased mobility of the left knee and swelling of the LLE.

**Figure 1:**
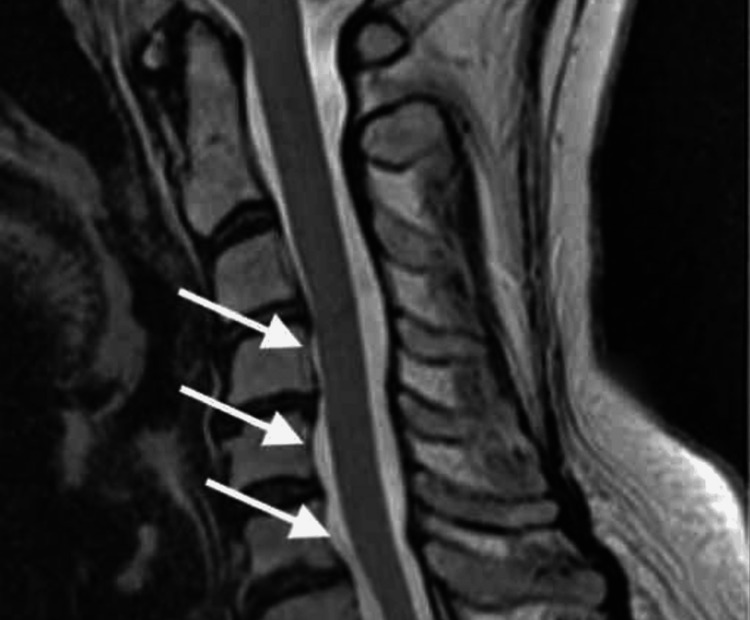
Cervical spine MRI MRI of the cervical spine demonstrating multilevel cervical spondylosis with moderate bilateral foraminal stenosis at C4-C6 (arrows). MRI: magnetic resonance imaging

**Figure 2:**
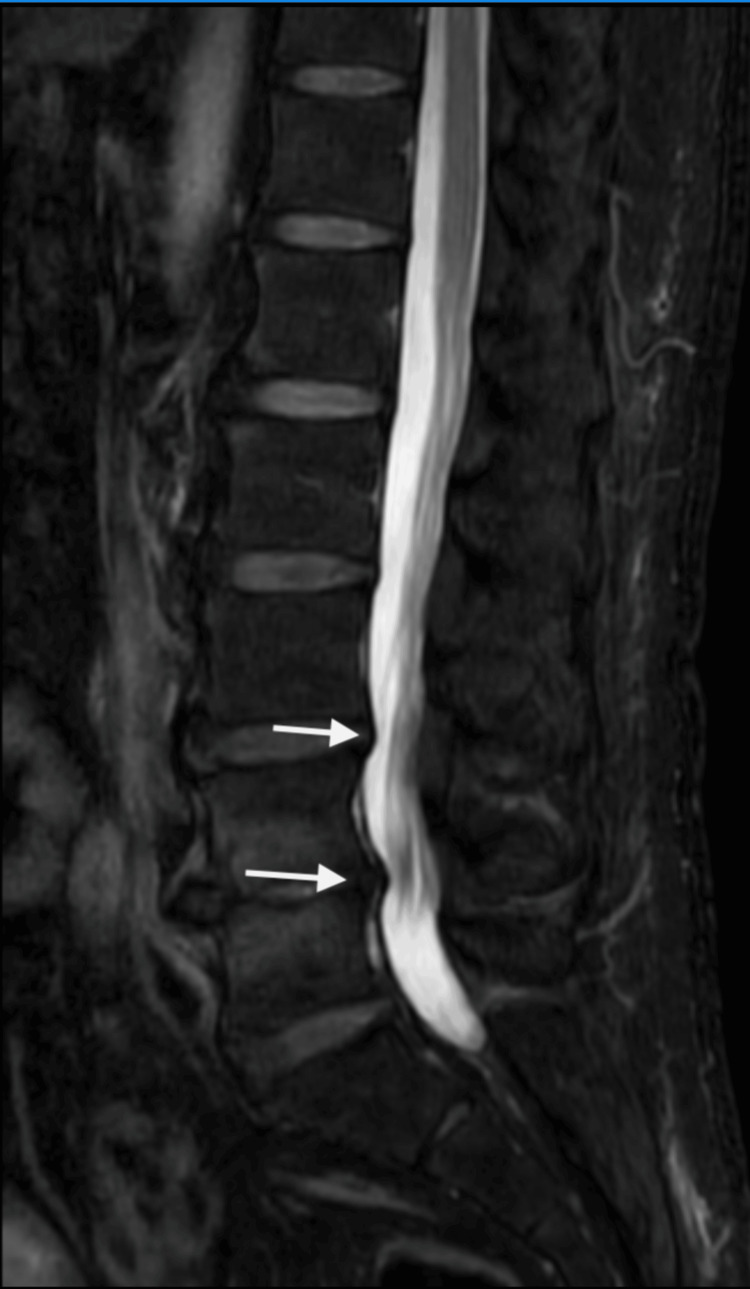
Lumbar spine MRI MRI of the lumbar spine showing moderate spinal stenosis at L3-L4 and L4-L5 (arrows). MRI: magnetic resonance imaging

**Figure 3:**
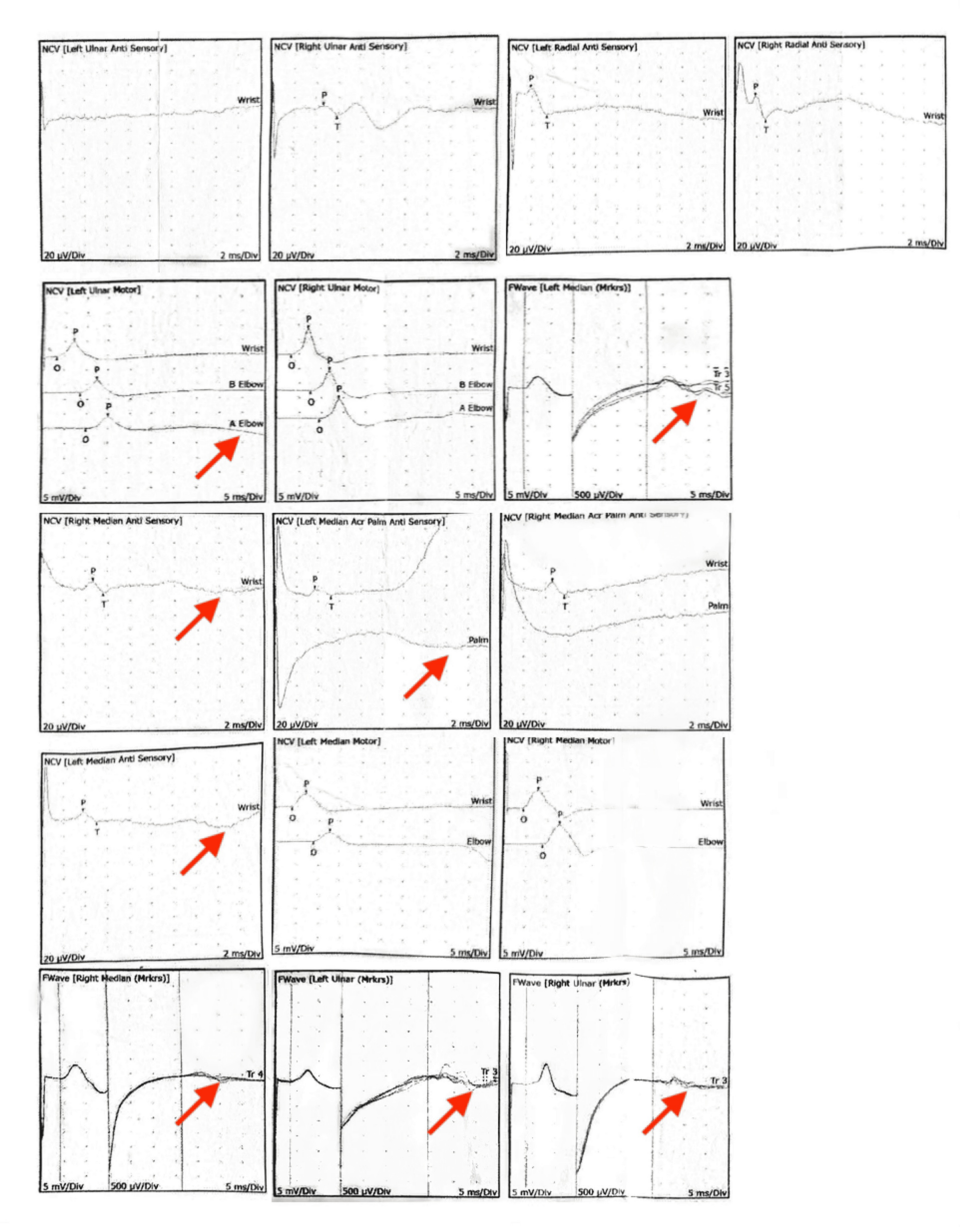
EMG of bilateral upper extremities Needle EMG was performed using sterile concentric needle electrodes to assess insertional activity, spontaneous activity, motor unit potentials, and recruitment patterns in clinically relevant muscles, following standard electrophysiological protocols. EMG of the upper extremities portrays chronic moderate left C5 and C6-C7 radiculopathy with axonal loss involving the left ulnar motor nerve, as well as bilateral mild to moderate median and ulnar neuropathies (arrows). EMG: electromyography

**Figure 4:**
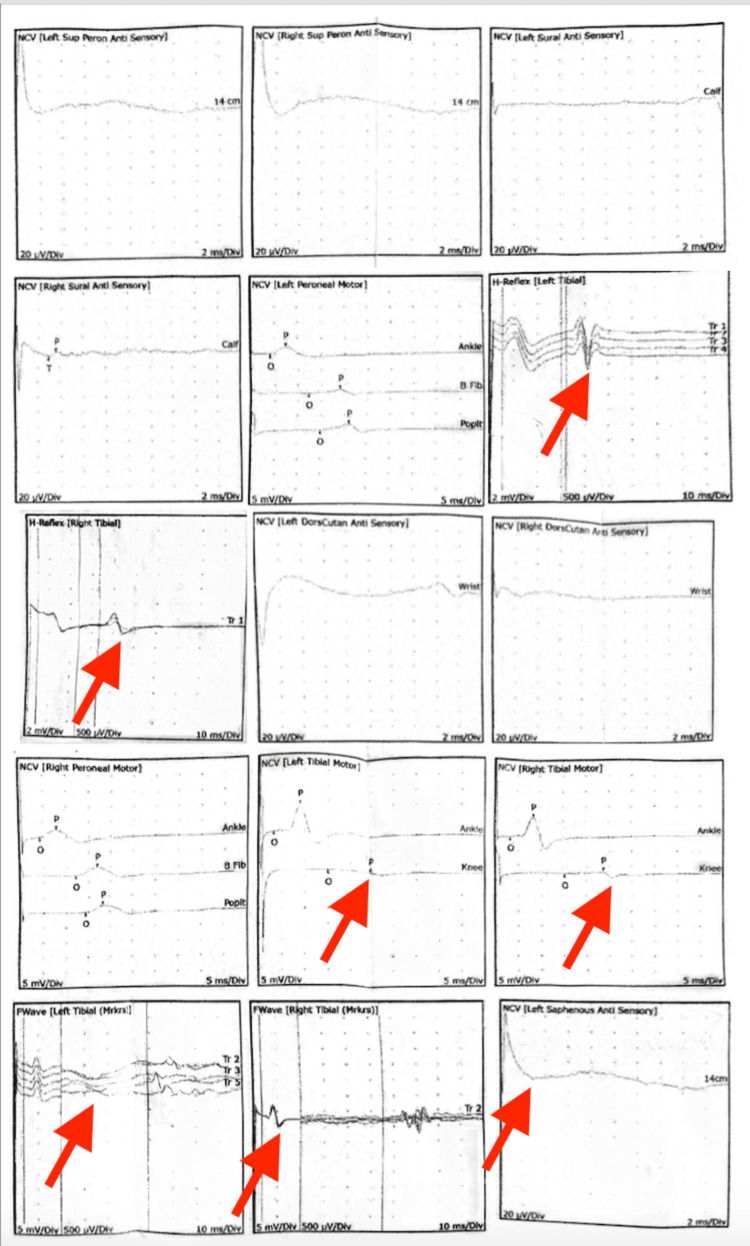
EMG of bilateral lower extremities EMG of the lower extremities revealed chronic bilateral L5-S1 radiculopathies, along with mild to moderate bilateral tibial and peroneal neuropathies (arrows). EMG: electromyography

Neurologic examination revealed allodynia and hyperalgesia in a C7 dermatomal distribution, decreased grip strength, and restricted shoulder mobility. Trophic changes and edema were noted in the LUE and LLE without signs of infection or thrombosis. Imaging and electrodiagnostic studies demonstrated multilevel cervical spondylosis with moderate bilateral foraminal stenosis at C4-C6 (Figure 1) and moderate spinal stenosis at L3-L4 and L4-L5 (Figure 2), as shown on cervical and lumbar spine MRI, respectively. Flexion and extension radiographs demonstrated no evidence of spinal instability. Deep venous thrombosis was excluded via duplex ultrasonography. Electromyography (EMG) of the upper extremities showed chronic moderate left C5 and C6-C7 radiculopathy with axonal loss involving the left ulnar motor nerve, as well as bilateral mild to moderate median and ulnar neuropathies (Figure 3). These findings were consistent with overlapping neuropathies due to cervical radiculopathy, carpal tunnel syndrome, and cubital tunnel syndrome. EMG of the lower extremities revealed chronic bilateral L5-S1 radiculopathies, along with mild to moderate bilateral tibial and peroneal neuropathies (Figure 4).

The constellation of chronic burning pain, regional edema, sensory disturbances, and motor impairment in the absence of ongoing malignancy or infection was consistent with complex regional pain syndrome (CRPS). The confirmed nerve injury, including radiculopathy, trauma, and postradiation changes, supported a diagnosis of CRPS type II. He received a series of cervical epidural steroid injections over the course of several months and a cortisone injection to the left gluteal region, each offering transient relief.

## Discussion

Complex regional pain syndrome (CRPS) is a clinical diagnosis established by using the Budapest criteria, requiring ongoing pain disproportionate to any inciting event, the presence of at least one symptom in three of four categories (sensory, vasomotor, sudomotor/edema, and motor/trophic), and at least one sign in two or more of these categories on physical examination [2]. This patient met all diagnostic criteria by presenting with sensory symptoms such as hyperesthesia and allodynia; vasomotor findings of asymmetric swelling and skin color changes; sudomotor involvement with edema of the left arm and leg; and motor/trophic features such as weakness, fasciculations, and reduced range of motion (ROM). In this case report, diagnostic clarity was obscured by multiple overlapping pathologies, including a history of trauma and malignancy. One possible mechanism is that injury to the CNS from thalamic metastases may have disrupted central pain processing pathways, thereby initiating or amplifying CRPS symptomatology. Simultaneously, peripheral nerve injury from cervical and lumbosacral radiculopathies could have provided a plausible anatomical substrate for CRPS type II. Additionally, the patient’s history of trauma and radiation therapy related to lung cancer treatment represents a well-documented risk factor for CRPS, further complicating the clinical picture.

It is important to differentiate CRPS from other disease states such as lymphedema, cellulitis, deep vein thrombosis (DVT), post-radiculopathy neuropathy, and paraneoplastic neuropathy. Lymphedema is a chronic condition caused by impaired lymphatic drainage and is characterized by non-pitting edema that is usually painless and lacks the autonomic or sensory abnormalities seen in CRPS [3]. Our patient did not undergo cancer-related lymphatic resection, reducing the likelihood of lymphedema. Cellulitis is a bacterial skin infection associated with localized erythema, warmth, tenderness, and systemic signs such as fever and leukocytosis, all of which were absent in this case [4]. DVT can cause unilateral swelling and discomfort but lacks the sensory and autonomic disturbances typical of CRPS; it was excluded via duplex ultrasonography [5]. Post-radiculopathy neuropathy typically presents with dermatomal sensory loss and weakness secondary to nerve root compression but does not usually feature vasomotor or sudomotor disturbances or limb edema [6]. Lastly, paraneoplastic neuropathy is a rare, immune-mediated condition linked to malignancy that presents as a diffuse, symmetric sensorimotor neuropathy rather than localized pain with autonomic features. It progresses more gradually and is not associated with localized edema [7]. A comprehensive paraneoplastic antibody panel was obtained and returned negative, effectively ruling out paraneoplastic syndromes. Additionally, EMG findings did not demonstrate the characteristic facilitation response, making Lambert-Eaton myasthenic syndrome unlikely.

Compared to cases in the existing literature, this patient’s presentation is relatively unique in that CRPS developed in the context of both central (thalamic metastases) and peripheral (cervical and lumbar radiculopathies) nervous system injuries, along with a history of trauma and radiation exposure [8]. While CRPS following CNS injury, such as stroke, has been described, few case reports detail its emergence in the setting of metastatic brain lesions, particularly with such extensive overlap of neuropathic and inflammatory contributors [9]. Most published cases involve isolated limb trauma or post-surgical changes as the primary triggers [10]. Additionally, CRPS involving multiple extremities, as seen in this patient’s left upper and lower limbs, is uncommon and further adds complexity to the diagnostic process.

In our case, multimodal therapy was initiated with the goal of improving function and quality of life. This included physical therapy for desensitization and ROM preservation, neuropathic agents such as gabapentin and duloxetine, and interventional pain management via sympathetic nerve blocks. Psychological support was also provided to help the patient develop chronic pain coping strategies, acknowledging the significant role of biopsychosocial factors in CRPS management. This comprehensive approach is consistent with recommendations in the literature, which emphasize early and aggressive multidisciplinary intervention [1]. CRPS has a poor prognosis when not treated promptly, and delays in diagnosis or treatment can lead to chronic pain and long-term disability [11]. Therefore, early involvement of pain specialists is crucial.

Despite appropriate multimodal treatment, the patient’s outcome was limited by the presence of cancer-related CNS disease, which adds significant complexity to recovery. Central sensitization due to metastatic thalamic involvement likely contributed to persistent pain and impaired functional gains. While some stabilization of symptoms and modest functional improvement were achieved, outcomes were not as favorable as those reported in patients without active malignancy or CNS involvement [8]. In contrast to literature showing improved prognosis with early intervention and limited comorbidity, this case underscores how overlapping central and peripheral pathologies, in the setting of advanced cancer, may restrict the effectiveness of even well-executed CRPS management plans [11].

In summary, this case illustrates a complex presentation of CRPS with contributions from both central and peripheral nervous system injury in a patient with metastatic lung cancer and a history of a fall. It highlights the importance of maintaining a high index of suspicion, utilizing the Budapest criteria, differentiating CRPS from mimicking conditions, and implementing early, individualized, and multidisciplinary treatment strategies [1]. Prompt recognition and intervention remain key, but in cases with extensive comorbidities, outcomes may be limited despite best practices.

## Conclusions

This case emphasizes the importance of considering CRPS in patients with chronic limb pain and swelling, especially in the setting of both central and peripheral neurologic insults. In our patient, a history of trauma, cervical and lumbar radiculopathies, cubital and carpal tunnel syndromes, and postradiation changes created diagnostic complexity that was compounded by CNS metastases. A paraneoplastic antibody panel and EMG findings were instrumental in excluding paraneoplastic syndromes and Lambert-Eaton myasthenic syndrome, ensuring diagnostic clarity. Timely diagnosis using the Budapest criteria, supported by electrodiagnostic studies and careful exclusion of mimicking conditions, remains critical to effective management. Early interdisciplinary intervention, including physical therapy, pharmacologic therapy, interventional procedures, and psychological support, can significantly improve outcomes, although prognosis may be limited in patients with overlapping oncologic and neurologic disease.

This case also underscores the need for heightened awareness of CRPS in oncology patients, where pain syndromes may be mistakenly attributed solely to malignancy or its treatment. Further research into CRPS in cancer-related populations is warranted to better characterize its mechanisms, refine diagnostic strategies, and optimize treatment approaches. Recognizing atypical presentations and initiating prompt, tailored care remain essential for reducing long-term pain and disability in this vulnerable population.
